# Effects of N-Acetylneuraminic Acid on Intestinal Microbial Composition and Metabolic Activity in a Piglet Model

**DOI:** 10.3390/vetsci13030295

**Published:** 2026-03-21

**Authors:** Jiaqi Zhang, Runhan Ye, Xuan Li, Xiaoyi Liu, Kaifeng Lian, Ran Xu, Yahui Chen, Weiyun Zhu, Kaifan Yu

**Affiliations:** 1 Laboratory of Gastrointestinal Microbiology, Jiangsu Joint International Research Laboratory of Animal Gastrointestinal Genomes, College of Animal Science and Technology, Nanjing Agricultural University, Nanjing 210095, China; 2022205046@stu.njau.edu.cn (J.Z.); yerunhan@stu.njau.edu.cn (R.Y.); 2020205034@stu.njau.edu.cn (X.L.); liuxiaoyi@stu.njau.edu.cn (X.L.); 2025105081@stu.njau.edu.cn (K.L.); xuran@stu.njau.edu.cn (R.X.); chenyahui@stu.njau.edu.cn (Y.C.); zhuweiyun@njau.edu.cn (W.Z.); 2 State Key Laboratory of Meat Quality Control and Cultured Meat Development, Nanjing Agricultural University, Nanjing 210095, China; 3 National Center for International Research on Animal Gut Nutrition, Nanjing Agricultural University, Nanjing 210095, China

**Keywords:** Neu5Ac, intestinal microbiota, acetate, formate, piglets

## Abstract

This study investigates the effects of N-acetylneuraminic acid (Neu5Ac) on intestinal microbial composition and metabolic activity in piglets using two complementary approaches: in vitro fermentation and in vivo dietary supplementation with coated Neu5Ac. In vitro fermentation results demonstrated that Neu5Ac stimulates higher production of formate and acetate by piglet intestinal microbiota compared with other human milk-derived monosaccharides (*p* < 0.05). In vivo feeding trials showed that dietary coated Neu5Ac significantly increased microbial α-diversity and altered the overall microbial composition in both the jejunum and colon (*p* < 0.05). For instance, coated Neu5Ac reduced the relative abundances of *ASV1 Clostridium* and *ASV17 Clostridium* in the jejunum, while raising the relative abundances of *ASV3 Veillonella*, *ASV4 Veillonella*, *ASV7 Lactobacillus salivarius*, *ASV11 Actinobacillus porcitonsillarum* in the jejunum, and *ASV41 Xylanibacter* in the colon (*p* < 0.05). Furthermore, coated Neu5Ac significantly elevated formate and acetate concentrations in the jejunum (*p* < 0.05) and exhibited a trend toward increased acetate levels in the colon (0.05 < *p* < 0.1). Collectively, using piglets as a model, this study demonstrates that Neu5Ac facilitates the intestinal colonization of beneficial microbes (e.g., *Lactobacillus*), leading to enhanced production of microbial metabolites, particularly formate and acetate, which may contribute to improved gut homeostasis in early life.

## Simple Summary

N-Acetylneuraminic acid (Neu5Ac), a major component of human milk oligosaccharides, is abundant in breast milk and plays a critical role in shaping the infant gut microbiota. Nevertheless, the effects of Neu5Ac on microbial composition and metabolic activity in both the small and large intestines during early life are still poorly understood. In this study, we first compared the fermentability and metabolite profiles of five milk-derived monosaccharides (glucose, galactose, fucose, Neu5Ac, and GlcNAc) using in vitro fermentation with piglet jejunal and colonic microbiota. We then examined the effects of dietary coated Neu5Ac supplementation on microbial composition and metabolic activities in the jejunum and colon of piglets. Our results indicate that Neu5Ac promotes the intestinal colonization of beneficial bacteria such as *Lactobacillus* and increases the production of microbial metabolites, notably formate and acetate, in piglets. These findings indicate that coated Neu5Ac is a promising additive for improving intestinal microbiota homeostasis in piglets.

## 1. Introduction

Human milk oligosaccharides (HMOs) are recognized as the third most abundant nutrient in breast milk after proteins and lipids, typically composed of five monosaccharides: glucose, galactose, N-acetylneuraminic acid (Neu5Ac), fucose, and N-acetylglucosamine (GlcNAc) [1,2]. These monosaccharides are linked via diverse glycosidic bonds to form structurally complex HMOs with a wide range of biological functions [2]. In the intestinal tract, Neu5Ac and fucose are both critical components of mucin glycans secreted by intestinal epithelial cells [2]. Localized at the terminal positions of glycans, they can directly interact with the gut microbiota and participate in regulating intestinal epithelial barrier function [3,4,5,6]. GlcNAc is likewise an essential component of mucin glycans, serving as the core initiating unit of glycan structures and playing a vital role in maintaining the integrity of the intestinal epithelial barrier [2,7]. Collectively, these monosaccharides provide abundant substrates for microbial growth in the mammalian gut, thereby playing a crucial role in gut microbiota colonization and intestinal epithelial development during early life [2,8].

Early-life microbial colonization plays a critical role in shaping the developing microbiota and supporting long-term host health [9,10]. In recent years, fucose, Neu5Ac, and GlcNAc have garnered increasing attention due to their crucial roles in regulating the gut microbiota and host physiology. For example, Duan et al. have indicated that fucose accelerates intestinal stem cell-mediated intestinal epithelial development by promoting *Akkermansia*-associated propionate metabolism [11]. Similarly, GlcNAc treatment has been shown to alleviate colitis symptoms and inhibit disease progression in mice by modulating intestinal mucosal T cell-mediated immune responses [12]. For Neu5Ac, it is known to be released and metabolized from sialylated HMOs (e.g., 3′-sialyllactose and 6′-sialyllactose) by key infant-associated gut bacteria such as *Bifidobacterium* and *Bacteroides* [4,13,14]. Nevertheless, the effects of dietary Neu5Ac on gut microbiota during early life remain poorly characterized.

Sialic acids are a family of sugars ubiquitously present on the surface of intestinal mucosa [3,15]. Among them, Neu5Ac is the most well-studied sialic acid [4,16,17]. Neu5Ac has been approved as a food ingredient in several countries and regions, including China, the European Union, Japan, Malaysia, and Singapore. As a nutritional substrate, Neu5Ac selectively promotes the colonization of specific gut microbes and stimulates the production of associated metabolites, such as acetate, butyrate, propionate, formate, and lactate [4,13,18]. These microbiota-derived metabolites have been shown to play crucial roles in regulating immune responses, energy metabolism, and intestinal development [19,20,21]. However, current research on Neu5Ac has largely focused on fecal microbiota of infants [17,22], limiting our understanding of microbial communities in other gastrointestinal regions—especially the small intestine.

To explore the effects of Neu5Ac on both the small and large intestinal microbiota and their metabolic activities during early life, we first examine the differences in fermentability and metabolite profiles of five milk-derived monosaccharides (glucose, galactose, fucose, Neu5Ac, and GlcNAc) using in vitro fermentation with jejunal and colonic microbiota from piglets. We subsequently focused on investigating the effects of dietary supplementation with coated Neu5Ac on microbial composition and metabolic activities in the jejunum and colon of piglets. The coated Neu5Ac was prepared using fluidized bed spray coating technology to achieve controlled release of Neu5Ac along the gastrointestinal tract [23]. Overall, this study aims to establish a basis for modulating the gut microbial community and metabolic activities through Neu5Ac administration, thereby offering potential strategies to promote intestinal health during early life.

## 2. Materials and Methods

### 2.1. Substrates

Glucose (purity ≥ 99.5%), galactose (purity ≥ 98%), fucose (purity ≥ 98%), Neu5Ac (purity ≥ 98%), and GlcNAc (purity ≥ 97%) were purchased from Aladdin Co., Ltd. (Shanghai, China).

### 2.2. Inoculum Preparation

Fresh jejunal and colonic digesta were collected from three male (Duroc × Landrace × Yorkshire) piglets with a body weight of 18.3 ± 1.04 Kg. The piglets were selected from different litters and had remained healthy with no clinical signs of disease since birth. All piglets were fed a commercial corn-soybean meal diet and had no exposure to antibiotics since birth. The jejunal digesta from the three piglets were pooled and homogenized. An aliquot of the homogenate was diluted 1:10 (*w*/*v*) with sterile phosphate-buffered saline (PBS). Colonic digesta was processed using the same protocol. After dilution, the digesta mixture was filtered through four layers of sterile gauze (pore size: 150 mesh; Biosharp Co., Ltd., Heze, China) to obtain jejunal and colonic inocula. All procedures were performed under anaerobic conditions in an anaerobic workstation (GeneScience E500, Wilmington, DE, USA).

### 2.3. Medium Preparation and Sample Collection

The basal medium was prepared as described in previously reported methods [24]. Briefly, the medium contained (per liter) the following components: 0.6 g KCl, 1.46 g KH_2_PO_4_, 3.55 g Na_2_HPO_4_, 0.6 g NaCl, 0.5 g MgSO_4_·7H_2_O, 0.2 g CaCl_2_·2H_2_O, 1 mL resazurin, 10 mL trace elements, 1.0 g trypticase, 1 mL vitamin phosphate, 0.54 g NH_4_Cl, 10 mL hemin, 50 mL NaHCO_3_ solution. After boiling, cysteine-HCl (1 g/L) was added, and the pH was adjusted to 6.8~7.0. Each 250 mL serum bottle was filled with 90 mL of basal medium and flushed with CO_2_ to create a 160 mL headspace. Glucose, galactose, fucose, Neu5Ac, and GlcNAc were added to a final concentration of 1% (*w*/*v*) for batch-culture incubation. Following autoclaving, 1 mL of vitamin solution was added to each serum bottle containing the basal medium and monosaccharide. For inoculation, 10 mL of jejunal or colonic inoculum was added to the respective bottles. Incubation was carried out at 37 °C with shaking at 100 rpm for 48 h.

The experiment consisted of six treatment groups, each with four replicates (one serum bottle per replicate): a blank control (no monosaccharide), glucose (GLU), galactose (GAL), fucose (FUC), Neu5Ac, and GlcNAc groups. Fermentation broth samples were collected at 0, 6, 12, 24, and 48 h of incubation. pH was measured using a calibrated pH meter (Orion Star^TM^ A321, Thermo Fisher Scientific, Waltham, MA, USA). Samples for subsequent analysis were stored at −80 °C. Gas production was measured using a pressure transducer method as previously described [25]. Gas production volume was recorded every 6 h. After each measurement, gas was released to return the bottle pressure to atmospheric level (0 kPa) to ensure accuracy in subsequent measurements. Total gas production was calculated by summing the gas volumes recorded at each 6 h interval.

### 2.4. DNA Extraction and Real-Time Quantitative PCR

Total genomic DNA was extracted from 48 h fermentation broth samples using the QIAamp^®^ DNA Stool Mini Kit (QIAGEN, Hilden, Germany). The gene copies of total bacteria were quantified via quantitative polymerase chain reaction (qPCR) on a QuantStudio^TM^ 7 Flex Real-Time PCR system (Thermo Fisher Scientific, Waltham, MA, USA) with SYBR^®^ Green dye (Takara Bio Inc., Kusatsu, Shiga, Japan). Plasmid standards for total bacteria were prepared following our previously established protocol [26]. Copy numbers were calculated using standard curves generated from ten-fold serial dilutions of linearized plasmid DNA. Primer sequences for total bacteria were referenced from a previous study [27] and are listed in Supplemental Table S1.

### 2.5. Coated N-Acetylneuraminic Acid Treatment Experiments in Piglets

A total of 36 male Duroc × Landrace × Yorkshire piglets (21 d old) with similar initial body weight (5.82 ± 0.06 kg) were randomly assigned to two dietary groups. Each group contained six pens, with three piglets per pen. The control group received a basal diet, and the Neu5Ac group received the same basal diet supplemented with coated Neu5Ac at 3 g/kg (purity = 30%). The selection of the Neu5Ac concentration was based on the studies by Yang et al. and Wang et al. [28,29]. The coated Neu5Ac was produced by Hangzhou King Techina Technology Co., Ltd. (Hangzhou, China) using intelligent microencapsulation technology, which facilitates passage through the stomach and controlled release along the intestinal tract. All piglets had free access to feed and water in accordance with standard pig farm management practices. The composition and nutrient levels of the basal diet in this study were based on the protocol described by Fan et al. [30]. On day 15 of the trial, following an 8 h fast, one piglet with a body weight closest to the pen average was selected from each pen for euthanasia, resulting in six biological replicates per group (*n* = 6). The specific procedure for euthanasia was as follows: piglets were first electrically stunned by exposure to a current of 200~300 V at a frequency of 50–60 Hz for at least 3 s [31]. After ensuring complete loss of consciousness, the animals were euthanized by exsanguination. Mid-jejunal and mid-colonic digesta samples were collected, snap-frozen in liquid nitrogen, and stored at −80 °C for subsequent 16S rRNA gene sequencing and metabolite analysis.

### 2.6. Acetate, Propionate, Butyrate, Formate, and Lactate Analysis

Concentrations of acetate, propionate, and butyrate were measured by gas chromatography following a previously reported method [25]. Formate and lactate concentrations were quantified via high-performance liquid chromatography (HPLC, ThermoFisher Scientific, Waltham, MA, USA) as previously described [26,32]. Briefly, 0.5 mL of fermentation broth or 0.5 mL of intestinal digesta suspension (digesta:purified water = 1:1, *w*/*v*) was thoroughly mixed with 100 μL of 25% (*w*/*v*) metaphosphoric acid-crotonic acid solution and stored overnight at −20 °C. After thawing, the mixtures were centrifuged at 12,000 rpm for 15 min. The resulting supernatant was collected and filtered through a 0.22 μm membrane filter. Acetate, propionate, and butyrate contents in the filtered solution were determined using a gas chromatograph (Shimadzu Corporation, Kyoto, Japan) equipped with a Nukol capillary column (30 m × 0.32 mm × 0.25 μm) (Sigma Co. Ltd., St. Louis, MO, USA). Formate and lactate contents were analyzed using an HPLC system (Ultimate 3000, ThermoFisher Scientific, Waltham, MA, USA).

### 2.7. DNA Extraction and 16S rRNA Gene Sequencing

Total genomic DNA was extracted from the jejunal and colonic digesta of 12 piglets using a DNA stool Mini Kit (QIAGEN, Hilden, Germany). DNA concentrations were quantified with a NanoDrop 2000 (Thermo Fisher Scientific, Waltham, MA, USA), and DNA integrity was assessed via 1% agarose gel electrophoresis. The V3–V4 hypervariable regions of the bacterial 16S rRNA gene were amplified by PCR using the universal primer pair 341F (5′-ACTCCTACGGGAGGCAGCAG-3′) and 806R (5′-GGACTACHVGGGTWTCTAAT-3′). Amplicons were purified using an Axygen DNA Gel Extraction Kit (Axygen Biosciences, Union City, CA, USA) and subjected to paired-end sequencing on an Illumina HiSeq 2500 platform following the manufacturer’s standard protocols. Raw sequence data were demultiplexed and quality-filtered using the Quantitative Insights Into Microbial Ecology 2 (QIIME 2) pipeline (version 2020.2) [33]. Amplicon Sequence Variants (ASVs) were resolved at 100% sequence similarity using DADA2 (version 1.18.0) [34]. Taxonomic classification of ASVs was performed using the Ribosomal Database Project (RDP) Classifier (version 2.2) against the Silva reference database (version 138), with a confidence threshold of 70%. Alpha diversity (Chao1 index) of the microbial community was calculated using QIIME 2. Beta diversity was visualized via principal coordinate analysis (PCoA) based on the Bray–Curtis dissimilarity matrix, which quantifies compositional dissimilarity between samples.

### 2.8. Statistical Analysis

All data were analyzed using SPSS 20.0 software (SPSS Inc., Chicago, IL, USA). For microbiome data, differences in the Chao1 and Shannon index and bacterial abundance (at the phylum, genus, and ASV levels) among groups were assessed using the Mann–Whitney U test due to the non-normal distribution, and the results were expressed as medians. To mitigate the false positive rate inherent in the large-scale microbiome dataset, a false discovery rate (FDR) correction was applied using the Benjamini–Hochberg method. Statistical significance was set at an adjusted *p* value of <0.05. For the in vitro fermentation parameters (gas production, pH, and levels of acetate, propionate, butyrate, formate, and lactate), differences among multiple groups over time were assessed using a two-way repeated measures ANOVA. When a significant interaction or main effect was detected, post hoc multiple comparisons were conducted. In the in vivo trial, an independent sample t-test was employed to compare metabolite concentrations (acetate, propionate, butyrate, formate, and lactate) between the CON and Neu5Ac groups. For the fermentation and metabolite data, results are presented as mean ± standard error (SEM).

## 3. Results

### 3.1. pH, Total Gas Production, and Total Bacterial Abundance During In Vitro Fermentation

**Figure 1:**
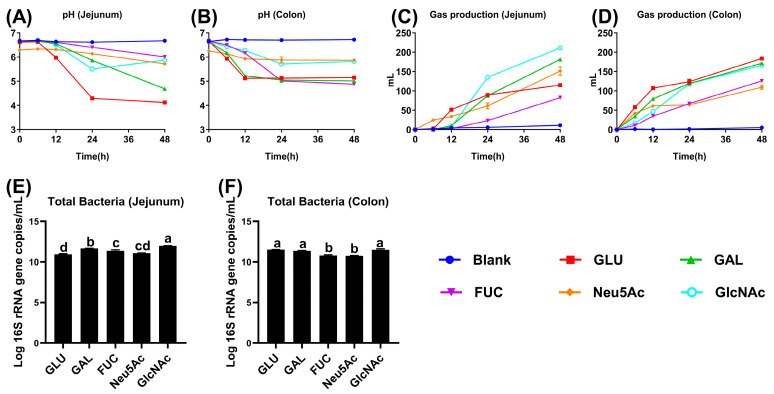
Total gas production, fermentation broth pH, and total bacterial abundance during in vitro fermentation of jejunal and colonic microbiota from piglets with human milk-derived monosaccharide. (**A**) pH of jejunal fermentation broth. (**B**) pH of colonic fermentation broth. (**C**) Total gas production of the jejunal fermentation broth. (**D**) Total gas production of the colonic fermentation broth. (**E**) The gene copy number of the total bacteria in the jejunal fermentation broth. (**F**) The gene copy number of the total bacteria in the colonic fermentation broth. Data are expressed as means ± SEM (*n* = 4). ^a,b,c,d^ Mean that values with different superscripts in the same row are significantly different (*p* < 0.05). GLU, glucose; GAL, galactose; FUC, fucose; Neu5Ac, N-acetylneuraminic acid; GlcNAc, N-acetylglucosamine.

During the in vitro fermentation, total gas production and fermentation broth pH were measured at 0, 6, 12, 24, and 48 h. In jejunal fermentation, the GLU group exhibited the lowest pH at 12, 24, and 48 h among all groups (*p* < 0.05, Figure 1A and Table S2). In colonic fermentation, pH values in the GLU, GAL, and FUC groups were significantly lower at 24 and 48 h compared to the other groups (*p* < 0.05, Figure 1B and Table S2). Gas production analysis revealed that, in jejunal fermentation, the GLU, GAL, Neu5Ac, and GlcNAc groups produced more gas than the FUC group at 24 and 48 h, with the GlcNAc group exhibiting the highest gas production among all groups (*p* < 0.05, Figure 1C and Table S2). Similarly, in colonic fermentation, gas production in the GLU, GAL, and GlcNAc groups exceeded that in the FUC and Neu5Ac groups at 24 and 48 h (*p* < 0.05, Figure 1D and Table S2). The results of total bacterial quantification indicated that the GlcNAc group exhibited the highest total bacterial gene copy numbers in both jejunal and colonic fermentation broths compared to the other groups (*p* < 0.05, Figure 1E,F). Taken together, these results indicate that significant differences in total gas production and fermentation broth pH were observed during the in vitro fermentation of jejunal and colonic digesta supplemented with GLU, GAL, FUC, Neu5Ac, and GlcNAc. Specifically, GLU and GAL exhibited a pronounced ability to reduce pH and promote gas production, whereas GlcNAc demonstrated a strong ability to enhance total bacterial gene copy numbers.

### 3.2. Concentrations of Formate, Lactate, Acetate, Propionate, and Butyrate in Jejunal and Colonic Fermentation Broths

**Figure 2:**
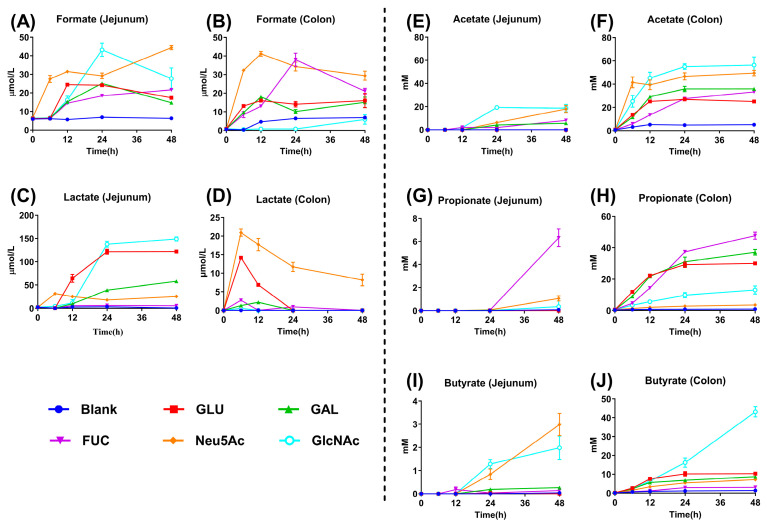
Production of formate, lactate, acetate, propionate, and butyrate during in vitro fermentation of piglet jejunal and colonic microbiota with human milk-derived monosaccharides. (**A**–**J**) The formate, lactate, acetate, propionate, and butyrate concentrations in the jejunal and colonic fermentation broth. Data are expressed as means ± SEM (*n* = 4). GLU, glucose; GAL, galactose; FUC, fucose; Neu5Ac, N-acetylneuraminic acid; GlcNAc, N-acetylglucosamine.

The concentrations of formate, lactate, acetate, propionate, and butyrate were determined in jejunal and colonic fermentation broths at 0, 6, 12, 24, and 48 h. Compared with the GLU, GAL, and FUC groups, jejunal fermentation with Neu5Ac produced higher formate levels at 6, 12, 24, and 48 h (*p* < 0.05, Figure 2A and Table S3). Similarly, compared to the GLU, GAL, and GlcNAc groups, colonic fermentation with Neu5Ac also produced increased formate production at 6, 12, 24, and 48 h (*p* < 0.05, Figure 2B and Table S3). In jejunal fermentation, the GLU and GlcNAc groups exhibited higher lactate concentrations at 24 and 48 h compared with the other groups (*p* < 0.05, Figure 2C and Table S3). Additionally, the Neu5Ac group showed the highest lactate concentration in colonic fermentation broth at 6, 12, 24, and 48 h (*p* < 0.05, Figure 2D and Table S3). Relative to the GLU, GAL, and FUC groups, the Neu5Ac and GlcNAc groups had significantly higher acetate concentrations in jejunal fermentation at 24 and 48 h, as well as in colonic fermentation at 6, 12, 24, and 48 h (*p* < 0.05, Figure 2E,F and Table S4). For propionate, the FUC group displayed the highest concentration in jejunal fermentation at 48 h compared with the other groups (*p* < 0.05, Figure 2G and Table S4). In colonic fermentation at 12, 24, and 48 h, the GLU, GAL, and FUC groups exhibited higher propionate levels than the GlcNAc and Neu5Ac groups (*p* < 0.05, Figure 2H and Table S4). Regarding butyrate, Neu5Ac and GlcNAc showed higher levels in jejunal fermentation at 24 and 48 h compared with the other groups (*p* < 0.05, Figure 2I and Table S4), while GlcNAc produced the highest butyrate concentration in colonic fermentation at 24 and 48 h (*p* < 0.05, Figure 2J and Table S4). Therefore, these results indicate that Neu5Ac fermentation by jejunal and colonic microbiota yields higher production of formate and acetate compared with other monosaccharides.

### 3.3. Effects of Dietary Coated Neu5Ac on the Intestinal Microbial Structure and Composition in Piglets

**Figure 3:**
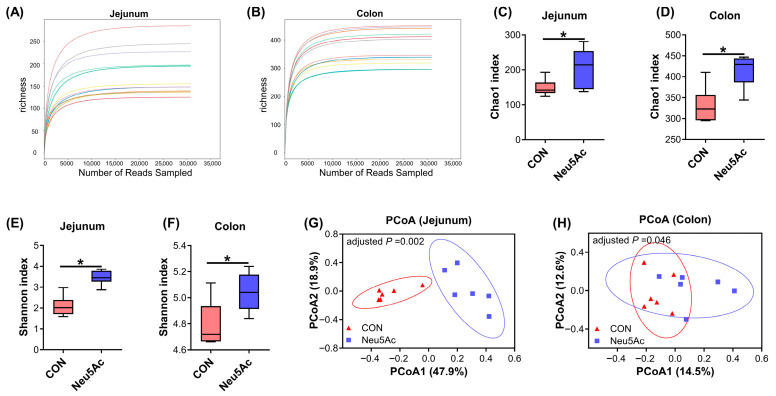
The effect of dietary coated Neu5Ac supplementation on gut microbial diversity in jejunal and colonic digesta of weaned piglets (*n* = 6). (**A**,**B**) The rarefaction curve of the jejunal and colonic microbiota. Different colored curves represent different samples. (**C**,**D**) The Chao1 index represents the α-diversity of the jejunal and colonic microbiota. (**E**,**F**) The Shannon index represents the α-diversity of the jejunal and colonic microbiota. (**G**,**H**) PCoA of microbiota in jejunal and colonic digesta based on the Bray–Curtis distance. * Adjusted *p* < 0.05. CON, piglets fed a basal diet; Neu5Ac, piglets fed a basal diet supplemented with coated Neu5Ac.

To further investigate the effects of Neu5Ac on the jejunal and colonic microbial structure and composition in piglets, a coated Neu5Ac feeding trial was conducted, and digesta samples from the jejunum and colon were collected for 16S rRNA sequencing analysis. In this study, a total of 24 intestinal digesta samples were sequenced. After quality control and assembly, 873,471 high-quality sequences were obtained, with an average of approximately 36,395 sequences per sample. The rarefaction curves of all jejunal and colonic samples have flattened, suggesting that the sequencing depth was sufficient for subsequent analyses (Figure 3A,B). For α-diversity analysis, dietary coated Neu5Ac significantly increased the Chao1 index and Shannon index of jejunal and colonic microbiota (*p* < 0.05, Figure 3C–F). PCoA revealed distinct overall microbial compositions between the CON and Neu5Ac groups in both the jejunum and colon (adjusted *p* < 0.05, Figure 3G,H).

**Figure 4:**
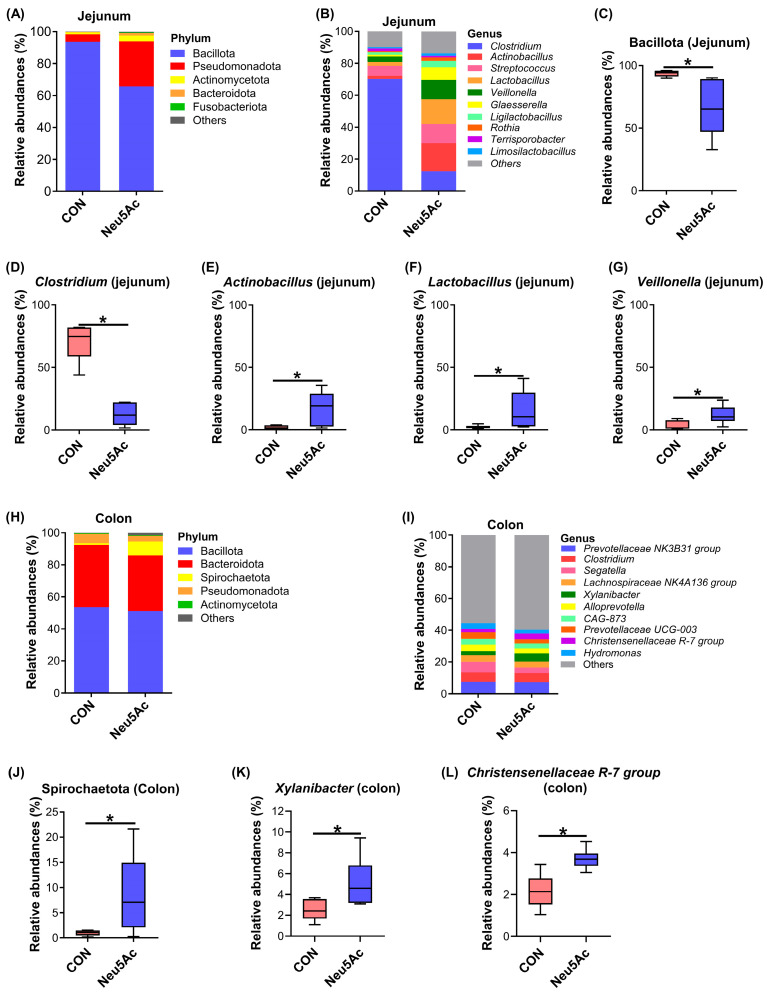
Effects of dietary coated Neu5Ac supplementation on gut microbial composition at the phylum and genus levels in jejunal and colonic digesta of weaned piglets. (**A**) Top 5 phylum-level distribution of jejunal microbiota (*n* = 6). (**B**) Top 10 genus-level distribution of jejunal microbiota. (**C**) Relative abundance of Bacillota in jejunal microbiota. (**D**–**G**) Relative abundances of differential microbiota at the genus level in jejunal microbiota, including (**D**) *Clostridium*, (**E**) *Actinobacillus*, (**F**) *Lactobacillus*, and (**G**) *Veillonella*. (**H**) Top 5 phylum-level distribution of colonic microbiota. (**I**) Top 10 genus-level distribution of colonic microbiota. (**J**) Relative abundance of Spirochaetota in colonic microbiota. (**K**,**L**) Relative abundances of differential microbiota at the genus level in colonic microbiota, including (**K**) *Xylanibacter*, and (**L**) *Christensenellaceae *R-7 group. * Adjusted *p* < 0.05. CON, piglets fed a basal diet; Neu5Ac, piglets fed a basal diet supplemented with coated Neu5Ac.

The predominant microbial phyla in the jejunum are shown in Figure 4A. Compared with the CON group, the Neu5Ac group exhibited a significantly lower relative abundance of Bacillota (*p* < 0.05, Figure 4C). At the genus level, the dominant taxa in the jejunum are illustrated in Figure 4B. Dietary supplementation with coated Neu5Ac significantly reduced the relative abundance of *Clostridium*, while markedly increasing the relative abundance of *Actinobacillus*, *Lactobacillus*, and *Veillonella* (*p* < 0.05, Figure 4D–G). The predominant microbial phyla and genera in the colon are displayed in Figure 4H,I. Notably, compared with the CON group, the Neu5Ac group showed a significant increase in the relative abundance of Spirochaetota, *Xylanibacter*, and the *Christensenellaceae* R-7 group (*p* < 0.05, Figure 4J–L).

**Figure 5:**
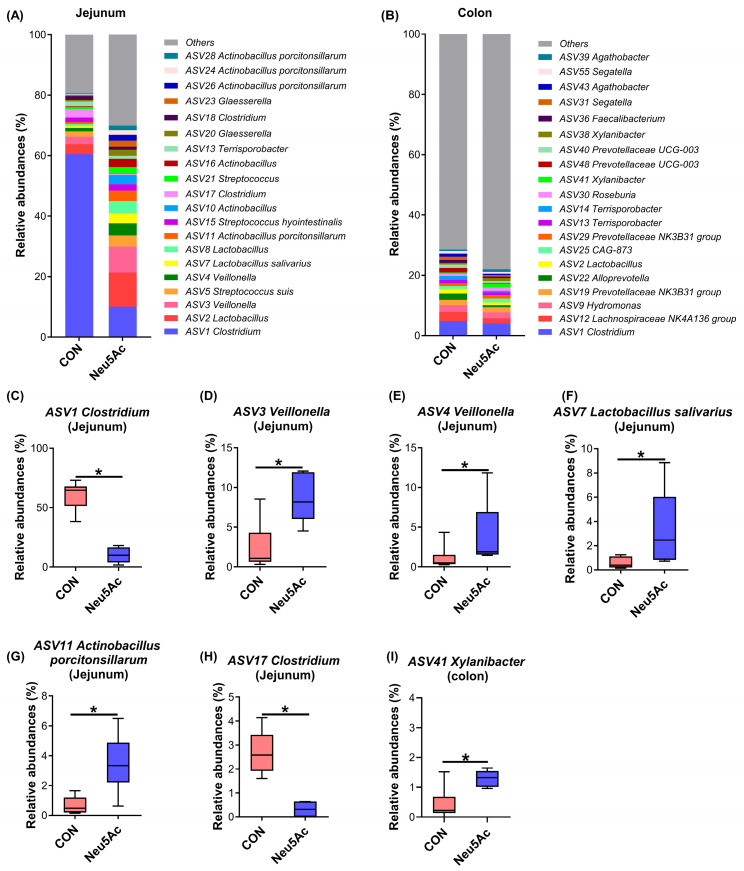
Effects of dietary coated Neu5Ac supplementation on gut microbial composition at ASV levels in jejunal and colonic digesta of weaned piglets (*n* = 6). (**A**,**B**) Top 20 microbial ASVs of jejunal and colonic microbia. Relative abundance of differential microbial ASVs in jejunal microbia, including (**C**) *ASV1 Clostridium*, (**D**) *ASV3 Veillonella*, (**E**) *ASV4 Veillonella*, (**F**) *ASV7 Lactobacillus salivarius*, (**G**) *ASV11 Actinobacillus porcitonsillarum*, and (**H**) *ASV17 Clostridium*. (**I**) Relative abundance of *ASV41 Xylanibacter* in colonic microbia. * *p* < 0.05. CON, piglets fed a basal diet; Neu5Ac, piglets fed a basal diet supplemented with coated Neu5Ac.

The predominant microbial ASVs in the jejunum and colon are presented in Figure 5A and Figure 5B, respectively. Compared with the CON group, the Neu5Ac group showed a significant decrease in the relative abundances of *ASV1 Clostridium* and *ASV17 Clostridium* in the jejunum (*p* < 0.05, Figure 5C,H). Conversely, the relative abundance of *ASV3 Veillonella*, *ASV4 Veillonella*, *ASV7 Lactobacillus salivarius*, and *ASV11 Actinobacillus porcitonsillarum* were significantly increased in the Neu5Ac group (*p* < 0.05, Figure 5D–G). In the colon, dietary Neu5Ac supplementation significantly increased the relative abundance of *ASV41 Xylanibacter* compared with the CON group (*p* < 0.05, Figure 5I). Collectively, these results demonstrate that dietary supplementation with coated Neu5Ac significantly altered the microbial structure and composition in both the jejunum and colon of piglets.

### 3.4. Effects of Dietary Coated Neu5Ac on the Concentrations of Formate, Lactate, Acetate, Propionate, and Butyrate in the Jejunum and Colon of Piglets

**Figure 6:**
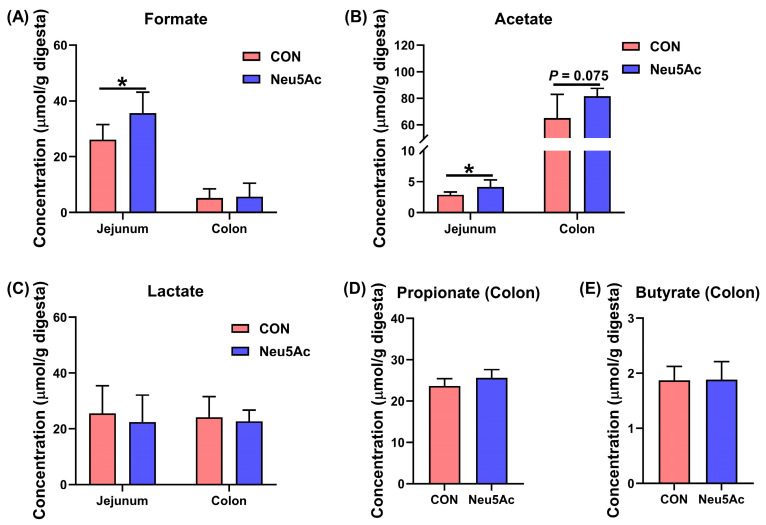
Effects of dietary coated Neu5Ac supplementation on formate, acetate, lactate, propionate, and butyrate concentrations in jejunal and colonic digesta of weaned piglets. (**A**–**C**) The concentration of formate, acetate, and lactate in the jejunum and colon. (**D**,**E**) The concentration of propionate and butyrate in the colon. Data are expressed as means ± SEM (*n* = 6). * *p* < 0.05. CON, piglets fed a basal diet; Neu5Ac, piglets fed a basal diet supplemented with coated Neu5Ac.

The concentrations of formate, lactate, acetate, propionate, and butyrate were measured in the jejunum and colon of piglets (Figure 6). Compared with the CON group, the Neu5Ac group exhibited significantly higher concentrations of formate and acetate in the jejunum, while acetate levels in the colon showed an increasing trend (*p* < 0.05, Figure 6A,B). In contrast, no significant differences were observed in colonic formate concentration or in the concentrations of lactate, propionate, and butyrate in jejunum and colon (*p* > 0.05, Figure 6C–E).

### 3.5. Correlation Analysis Between Metabolite Concentration and the Relative Abundance of the Top 20 Microbial ASVs

**Figure 7:**
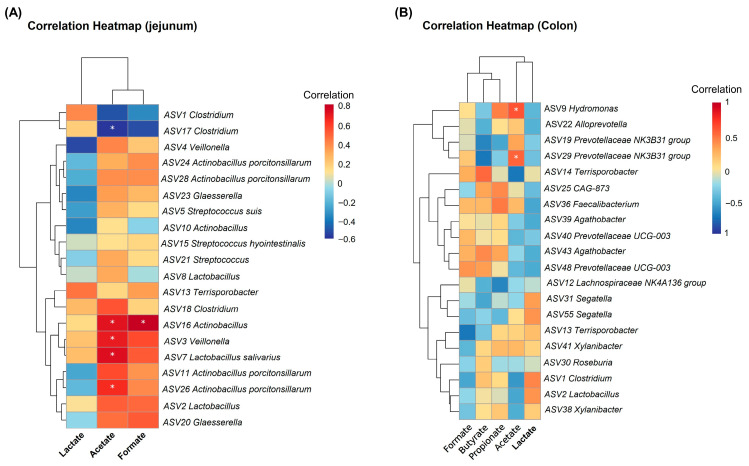
Correlation analysis between formate, lactate, acetate, propionate, and butyrate concentrations and the relative abundance of the top 20 microbial ASVs. (**A**) Correlation analysis between formate, acetate, and lactate concentrations and the relative abundance of the top 20 microbial ASVs in the jejunum (*n* = 6). (**B**) Correlation analysis between formate, lactate, acetate, propionate, and butyrate concentrations and the relative abundance of the top 20 microbial ASVs in the colon (*n* = 6). Spearman’s correlation coefficients were calculated, with red indicating positive correlations and blue indicating negative correlations. Darker colors represent stronger correlations. * Adjusted *p* < 0.05.

To further investigate which microbial taxa influenced changes in metabolite levels, correlation analysis was performed between the concentrations of formate, lactate, acetate, propionate, and butyrate and the relative abundances of the top 20 microbial ASVs. Correlation analysis in the jejunum revealed that acetate concentration was significantly positively correlated with the relative abundances of *ASV26 Actinobacillus porcitonsillaarum*, *ASV7 Lactobacillus salivarius*, *ASV3 Veillonella*, and *ASV16 Actinobacillus*, while showing a significant negative correlation with *ASV17 Clostridium* (adjusted *p* < 0.05, Figure 7A). Additionally, formate concentration was significantly positively correlated with the relative abundance of *ASV16 Actinobacillus* (adjusted *p* < 0.05, Figure 7A). In the colon, acetate concentration exhibited significant positive correlations with *ASV29 Prevotellaceae *NK3B31 group and *ASV9 Hydromonas* (*p* < 0.05, Figure 7B).

## 4. Discussion

Neu5Ac has recently been identified as a monosaccharide that modulates gut microbiota and host health [4,18]. Nevertheless, its effects on the gut microbial composition in early life remain largely unexplored. Based on our in vitro fermentation results, which showed that Neu5Ac stimulates the production of formate and acetate, we further conducted a feeding trial in piglets supplemented with coated Neu5Ac to validate these findings in vivo. Our results demonstrate that dietary coated Neu5Ac promotes a healthier gut microbial environment by facilitating the colonization of beneficial bacteria and enhancing the production of metabolites such as formate and acetate, underscoring its potential as a functional prebiotic for improving intestinal health in young animals.

Fucose, Neu5Ac, and GlcNAc are major components of HMOs, exhibiting unique benefits in promoting the colonization of beneficial bacteria and enhancing intestinal health [4,6,12]. Numerous studies have demonstrated that HMOs, as principal nutritional constituents of breast milk, exert a profound influence on the colonization and development of the gut microbiota in young animals during early life [30,35,36]. We therefore hypothesize that these milk-derived monosaccharides are functional components mediating the benefits of HMOs. To test this hypothesis, we used a piglet model to evaluate the effects of in vitro microbial fermentation of glucose, galactose, fucose, Neu5Ac, and GlcNAc in jejunal and colonic digesta from piglets on pH, gas production, the abundance of total bacteria, and the levels of formate, lactate, acetate, propionate, and butyrate. Fermentation of GLU, GAL, fucose, Neu5Ac, and GlcNAc yielded distinct profiles: GLU and GAL most effectively lowered pH and increased gas production, whereas GlcNAc most strongly stimulated total bacterial growth. These findings indicate that glucose, galactose, fucose, Neu5Ac, and GlcNAc can drive the growth of distinct microbial populations, with glucose and galactose being the most readily utilized monosaccharides by microbes, and GlcNAc being the most favorable for total bacterial growth.

Short-chain fatty acids (SCFAs), including acetate, propionate, and butyrate, along with formate and lactate, are key metabolites that support intestinal health and serve as indicators of prebiotic activity [20,21]. Notably, Neu5Ac fermentation yielded higher levels of formate and acetate than other monosaccharides in this study. Microbial-derived formate enters systemic circulation as a one-carbon unit, supporting nucleotide synthesis and methylation—processes vital in development, immunity, and disease [37]. Acetate, a major end-product of microbial fermentation, provides host energy and strengthens the intestinal barrier and immune function [38,39]. Furthermore, our results also showed that colonic fermentation of Neu5Ac yielded more lactate, while jejunal fermentation produced more butyrate relative to other monosaccharides. Collectively, these findings support the potential of Neu5Ac as a prebiotic that selectively promotes the colonization of SCFA-producing bacteria and may thereby enhance intestinal health.

Neu5Ac, positioned at the terminal ends of HMOs, contributes to host health by directly interacting with the gut microbiota [3,4]. The early-life period is a critical window for establishing host-microbiota interactions, where initial microbial colonization shapes the developing community and influences long-term host health [9,10]. To investigate changes in the gut microbiota during early life, this study employed a piglet model to study the effects of dietary supplementation with coated Neu5Ac on the microbial structure and function in the jejunum and colon. Coated Neu5Ac was synthesized using fluidized bed coating technology to reduce its degradation in the stomach and enable gradual release throughout the intestinal tract. This approach allowed us to examine the effects of Neu5Ac on the microbiota in both the small and large intestine. Our results showed that coated Neu5Ac significantly altered the overall microbial composition and increased the Chao1 index in both the jejunum and colon. Specifically, it reduced the relative abundances of *ASV1 Clostridium* and *ASV17 Clostridium* in the jejunum, while increasing the relative abundances of *ASV3 Veillonella*, *ASV4 Veillonella*, *ASV7 Lactobacillus salivarius*, and *ASV11 Actinobacillus porcitonsillarum* in the jejunum, as well as *Xylanibacter* and the *Christensenellaceae *R-7 group in the colon. These shifts suggest a targeted modulation of the gut microbial community. *Clostridium* is a dominant gut genus important for intestinal homeostasis [40], yet certain species *C. perfringens* and *C. difficile* are common diarrheal pathogens in piglets [41]. In the CON group, *Clostridium* was highly abundant (70.21 ± 14.48%), whereas Neu5Ac supplementation restored it to a low level (12.49 ± 8.47%). Due to the inherent resolution limitations of 16S rRNA gene sequencing, the specific *Clostridium* species reduced in the Neu5Ac group could not be definitively identified, and their potential pathogenicity or beneficial roles warrant further investigation. Conversely, several promoted bacteria are associated with beneficial functions. *Lactobacillus salivarius* is a well-characterized probiotic known to enhance barrier function and exert anti-inflammatory and metabolic effects [42,43,44]. *Veillonella*, *Xylanibacter*, and *the Christensenellaceae *R-7 group are recognized as beneficial bacteria involved in SCFA production in the gut [45,46,47]. Although *Actinobacillus porcitonsillarum* is commonly found in piglet intestines [48], its functional roles remain to be fully elucidated. Collectively, these findings indicate that dietary coated Neu5Ac appears to help maintain intestinal microbial homeostasis by reducing the abundance of potential diarrheal pathogens such as *Clostridium*, while promoting the growth of beneficial bacteria involved in SCFA production, including *Veillonella*, *Lactobacillus salivarius*, *Xylanibacter*, and *Christensenellaceae *R-7 group.

The development of the gut microbiota in early life has lifelong impacts on the host, and SCFAs serve as key metabolites mediating the interaction between the microbiota and the host. Formate, lactate, acetate, propionate, and butyrate are major products of carbohydrate fermentation by intestinal commensal bacteria and have been demonstrated to play essential roles in maintaining intestinal homeostasis [11,21,49,50]. In the present study, we examined the effects of dietary coated Neu5Ac supplementation on the levels of formate, lactate, acetate, propionate, and butyrate in the jejunum and colon. The results revealed that coated Neu5Ac significantly increased the concentrations of formate and acetate in the jejunum and exhibited a trend toward elevated acetate levels in the colon. These findings are consistent with our in vitro fermentation data, supporting the notion that Neu5Ac can drive the production of higher amounts of formate and acetate by jejunal and colonic microbiota in piglets. This observation is consistent with the broader role of milk-derived monosaccharides in shaping microbial metabolism. For example, Tsukuda et al. linked formate production to *Bifidobacterium* and showed that *B. infantis* and *B. breve* can generate formate from HMO-derived fucose [36]. Duan et al. reported that fucose treatment significantly increased the abundance of *Akkermansia* and the propionate metabolism in the mouse ileum, and also enhanced the production of acetate and propionate in the culture supernatant of *Akkermansia muciniphila* [11]. Similarly, our results demonstrate that Neu5Ac, another HMO monosaccharide, drives the intestinal microbiota toward higher formate and acetate production. However, the specific microbial taxa responsible for converting Neu5Ac to these metabolites in piglets remain unidentified. To explore this, we conducted correlation analyses between the concentrations of formate, lactate, acetate, propionate, and butyrate and the relative abundances of the top 20 ASVs. Acetate production showed strong positive correlations with several taxa, including *Actinobacillus porcitonsillarum*, *Lactobacillus salivarius*, *Veillonella*, *Actinobacillus*, *Clostridium*, *Hydrogenophaga*, and the *Prevotellaceae *NK3B31 group. Formate levels were closely associated with *Actinobacillus*. Similarly, Hashimoto et al. found that sialic acid metabolism is a distinctive feature of *Lactobacillus salivarius*, which shifts its fermentation profile from lactate to acetate when metabolizing 3′-sialyllactose [51], confirming that this species can utilize sialylated substrates like Neu5Ac to produce acetate. Although our data suggest a link between *Actinobacillus* and formate generation, the hypothesis that Neu5Ac promotes formate metabolism via *Actinobacillus* proliferation requires direct validation through future bacterial culture experiments. In summary, coated Neu5Ac appears to support intestinal homeostasis by enriching beneficial bacteria such as *Lactobacillus salivarius* and enhancing the production of formate and acetate. Our correlation analyses further point to specific bacterial candidates, particularly *Actinobacillus*, that may mediate these metabolic effects, offering mechanistic insight into how Neu5Ac modulates gut microbial function.

Additionally, several limitations of this study should be acknowledged. First, the in vitro fermentation experiment used pooled jejunal and colonic inoculum from three piglets. Although this approach minimizes individual variation and facilitates comparison of substrate-specific metabolic profiles—a common practice in fermentation studies—it may also mask inter-individual heterogeneity in microbial composition and metabolic capacity. Future studies using individual replicates are needed to elucidate host-specific responses to Neu5Ac and other milk-derived monosaccharides. Second, 16S rRNA gene sequencing, while widely used for microbial community analysis, has inherent resolution limitations that preclude taxonomic classification at the species level. Therefore, future research integrating metagenomic sequencing, culture-based isolation, and functional assays (e.g., metabolomic or transcriptomic analyses) is warranted to validate the proposed mechanisms and clarify the precise roles of these microbes in mediating the effects of Neu5Ac on intestinal homeostasis during early life.

## 5. Conclusions

In summary, our in vitro fermentation experiments showed that Neu5Ac stimulates formate and acetate production by piglet jejunal and colonic microbiota. Building on these findings, in vivo supplementation with coated Neu5Ac confirmed that its ability to promote the colonization of beneficial bacteria, such as *Lactobacillus*, and enhances the generation of their metabolites, particularly formate and acetate. These results provide experimental evidence supporting the potential of Neu5Ac as a targeted nutritional strategy to improve intestinal microbiota homeostasis in piglets.

## Data Availability

The original contributions presented in this study are included in the article/Supplementary Material. Further inquiries can be directed to the corresponding author(s).

## Supplementary Materials

The following supporting information can be downloaded at: https://www.mdpi.com/article/10.3390/vetsci13030295/s1, Table S1: Primer for total bacteria used in the present study; Table S2: Total gas production and fermentation broth pH during in vitro fermentation of jejunal and colonic microbiota from piglets with human milk-derived monosaccharide; Table S3: Production of formate and lactate during in vitro fermentation of piglet jejunal and colonic microbiota with human milk-derived monosaccharides; Table S4: Production of acetate, propionate, and butyrate during in vitro fermentation of piglet jejunal and colonic microbiota with human milk-derived monosaccharides.

## Abbreviations

The following abbreviations are used in this manuscript:

FUC	Fucose
GAL	Galactose
GlcNAc	N-acetylglucosamine
GLU	Glucose
HMO	Human milk oligosaccharides
Neu5Ac	N-acetylneuraminic acid
SCFA	Short-chain fatty acids
