# Supplementary material for: Effects of N-Acetylneuraminic Acid on Intestinal Microbial Composition and Metabolic Activity in a Piglet Model
Source: Vet Sci. 2026 Mar 21;13(3):295. doi: 10.3390/vetsci13030295 (PMC13030646; doi:10.3390/vetsci13030295)
Supplement: Supplementary file 1 [file vetsci-13-00295-s001.zip › vetsci-4180753-supplementary.pdf]

Table S1 Primer for total bacteria used in the present study

Items	Primer sequence (5'-3')	Annealing temperature (°C)
Total bacteria	F: ACTCCTACGGGAGGCAGCAG<br>R: ATTACCGCGGCTGCTGG	60

Table S2 Total gas production and fermentation broth pH during *in vitro* fermentation of jejunal and colonic microbiota from piglets with human milk-derived monosaccharide.

**Items[1]**	**GLU**	**GAL**	**FUC**	**Neu5Ac**	**GlcNAc**	***P* value**
**Jejunum**						
pH (0 h)	6.62±0.008^{a}	6.62±0.013^{a}	6.64±0.003^{a}	6.29±0.009^{b}	6.63±0.005^{a}	Time: $P < 0.01$; Treatment: $P < 0.01$; Time * Treatment: $P < 0.01$
pH (6 h)	6.63±0.003^{b}	6.65±0.011^{b}	6.67±0.005^{ab}	6.33±0.009^{c}	6.70±0.017^{a}	
pH (12 h)	5.98±0.043^{d}	6.56±0.013^{ab}	6.61±0.005^{a}	6.31±0.009^{c}	6.51±0.006^{b}	
pH (24 h)	4.29±0.009^{e}	5.86±0.007^{c}	6.40±0.014^{a}	6.14±0.012^{b}	5.51±0.017^{d}	
pH (48 h)	4.12±0.014^{d}	4.69±0.013^{c}	6.01±0.062^{a}	5.72±0.005^{b}	5.88±0.026^{a}	
GP (mL, 0 h)	0.00±0.000	0.00±0.000	0.00±0.000	0.00±0.000	0.00±0.000	Time: $P < 0.01$; Treatment: $P < 0.01$; Time * Treatment: $P < 0.01$
GP (mL, 6 h)	1.13±0.304^{b}	0.00±0.000^{b}	2.93±2.925^{b}	24.38±0.625^{a}	0.00±0.000^{b}	
GP (mL, 12 h)	51.50±2.598^{a}	10.05±0.166^{c}	2.25±1.315^{d}	33.50±1.323^{b}	7.00±1.080^{cd}	
GP (mL, 24 h)	89.53±0.504^{b}	87.40±1.856^{b}	23.00±1.429^{d}	61.10±3.749^{c}	135.18±0.614^{a}	
GP (mL, 48 h)	114.80±2.981^{d}	181.53±3.948^{b}	82.35±1.932^{e}	151.25±5.328^{c}	211.13±1.912^{a}	
**Colon (%)**						
pH (0 h)	6.67±0.010^{a}	6.64±0.007^{a}	6.66±0.007^{a}	6.26±0.006^{b}	6.65±0.005^{a}	Time: $P < 0.01$; Treatment: $P < 0.01$; Time * Treatment: $P < 0.01$
pH (6 h)	5.94±0.038^{c}	6.168±0.005^{b}	6.50±0.019^{a}	6.14±0.005^{b}	6.43±0.011^{a}	
pH (12 h)	5.12±0.009^{e}	5.23±0.009^{d}	6.16±0.013^{b}	5.93±0.015^{c}	6.27±0.041^{a}	
pH (24 h)	5.13±0.007^{b}	5.05±0.088^{b}	5.01±0.023^{b}	5.88±0.065^{a}	5.72±0.013^{a}	
pH (48 h)	5.15±0.025^{b}	5.01±0.008^{c}	4.88±0.025^{d}	5.87±0.025^{a}	5.82±0.013^{a}	
GP (mL, 0 h)	0.00±0.000	0.00±0.000	0.00±0.000	0.00±0.000	0.00±0.000	Time: $P < 0.01$; Treatment: $P < 0.01$; Time * Treatment: $P < 0.01$
GP (mL, 6 h)	57.63±1.897^{a}	34.13±2.453^{c}	10.50±0.500^{d}	42.25±1.974^{b}	16.50±1.190^{d}	
GP (mL, 12 h)	106.88±0.688^{a}	79.38±0.944^{b}	34.75±1.250^{e}	61.03±0.765^{c}	46.78±2.543^{d}	
GP (mL, 24 h)	123.75±6.142^{a}	119.13±2.295^{a}	67.25±2.136^{b}	63.75±0.479^{b}	118.63±2.982^{a}	
GP (mL, 48 h)	183.88±2.135^{a}	171.38±0.747^{b}	125.63±0.688^{c}	109.50±2.630^{d}	165.63±1.908^{b}	

GP, gas production.

[1]Data were expressed as mean ± standard error.

[a, b, c, d, e]Mean that values with different superscripts in the same row are significantly different ($P < 0.05$).

Table S3 Production of formate and lactate during *in vitro* fermentation of piglet jejunal and colonic microbiota with human milk-derived monosaccharides.

Items[1]	GLU	GAL	FUC	Neu5Ac	GlcNAc	*P* value
**Jejunum**						
Formate (μM, 0 h)	6.20±0.034	6.24±0.049	6.16±0.261	6.77±0.234	6.45±0.288	
Formate (μM, 6 h)	6.37±0.109^{b}	6.41±0.141^{b}	6.13±0.046^{b}	27.52±0.938^{a}	6.51±0.483^{b}	
Formate (μM, 12 h)	24.42±0.739^{b}	15.25±0.109^{c}	14.49±0.540^{c}	31.50±0.233^{a}	16.24±1.053^{c}	Time: *P* < 0.01; Treatment: *P* < 0.01; Time * Treatment: *P* < 0.01
Formate (μM, 24 h)	24.140±0.353^{c}	25.09±0.302^{c}	18.44±0.512^{d}	29.18±0.762^{b}	43.14±1.757^{a}	
Formate (μM, 48 h)	17.39±0.349^{cd}	14.73±0.289^{d}	21.64±0.717^{c}	44.36±0.553^{a}	27.70±2.877^{b}	
Lactate (μM, 0 h)	2.35±0.195	2.51±0.086	2.33±0.063	2.28±0.097	2.48±0.149	
Lactate (μM, 6 h)	0.37±0.031^{c}	0.60±0.019^{c}	0.58±0.096^{c}	31.42±0.728^{a}	4.526±0.906^{b}	
Lactate (μM, 12 h)	64.42±8.508^{a}	9.64±0.156^{bc}	5.74±0.935^{c}	25.49±0.393^{b}	11.39±3.566^{bc}	Time: *P* < 0.01; Treatment: *P* < 0.01; Time * Treatment: *P* < 0.01
Lactate (μM, 24 h)	121.60±5.220^{b}	38.91±2.103^{c}	6.178±0.656^{d}	17.96±0.462^{d}	137.55±3.200^{a}	
Lactate (μM, 48 h)	121.85±1.24^{b}	58.13±1.845^{c}	5.54±0.752^{e}	25.55±1.394^{d}	148.86±2.317^{a}	
**Colon (%)**						
Formate (μM, 0 h)	0.88±0.023	0.82±0.011	0.83±0.101	0.84±0.039	0.95±0.056	
Formate (μM, 6 h)	13.11±0.126^{b}	9.66±0.320^{c}	8.49±1.595^{c}	32.33±0.422^{a}	0.51±0.099^{d}	
Formate (μM, 12 h)	16.03±0.213^{b}	18.01±0.851^{b}	12.96±0.326^{c}	41.16±0.638^{a}	0.83±0.014^{d}	Time: *P* < 0.01; Treatment: *P* < 0.01; Time * Treatment: *P* < 0.01
Formate (μM, 24 h)	14.06±1.473^{b}	10.17±1.105^{b}	37.97±3.507^{a}	34.32±1.19^{a}	0.83±0.017^{c}	
Formate (μM, 48 h)	16.02±3.809^{bc}	15.10±2.807^{bc}	21.05±1.558^{ab}	29.40±1.202^{a}	5.88±1.272^{c}	
Lactate (μM, 0 h)	0.00±0.000	0.00±0.000	0.00±0.000	0.00±0.000	0.00±0.000	
Lactate (μM, 6 h)	14.17±0.275^{b}	1.30±0.476^{cd}	2.71±0.196^{c}	20.92±0.483^{a}	0.56±0.210^{d}	
Lactate (μM, 12 h)	6.86±0.327^{b}	2.17±0.203^{c}	0.00±0.000^{d}	17.69±0.832^{a}	0.00±0.000^{d}	Time: *P* < 0.01; Treatment: *P* < 0.01; Time * Treatment: *P* < 0.01
Lactate (μM, 24 h)	0.00±0.000^{b}	0.00±0.000^{b}	0.94±0.417^{b}	11.71±0.614^{a}	0.00±0.000^{b}	
Lactate (μM, 48 h)	0.00±0.000^{b}	0.00±0.000^{b}	0.00±0.000^{b}	8.17±0.781^{a}	0.00±0.000^{b}	

[1]Data were expressed as mean ± standard error.

[a, b, c, d, e]Mean that values with different superscripts in the same row are significantly

different ($P < 0.05$).

Table S4 Production of acetate, propionate, and butyrate during *in vitro* fermentation of piglet jejunal and colonic microbiota with human milk-derived monosaccharides.

Items[1]	GLU	GAL	FUC	Neu5Ac	GlcNAc	*P* value
**Jejunum**						
Acetate (mM, 0 h)	0.00±0.000	0.00±0.000	0.00±0.000	0.00±0.000	0.00±0.000	Time: $P < 0.01$; Treatment: $P < 0.01$; Time * Treatment: $P < 0.01$
Acetate (mM, 6 h)	0.00±0.000	0.00±0.000	0.00±0.000	0.00±0.000	0.00±0.000	
Acetate (mM, 12 h)	0.00±0.000^{b}	0.00±0.000^{b}	2.09±0.236^{a}	0.00±0.000^{b}	1.87±0.171^{a}	
Acetate (mM, 24 h)	0.00±0.000^{e}	4.10±0.486^{c}	1.95±0.255^{d}	6.24±0.579^{b}	19.23±0.579^{a}	
Acetate(mM, 48 h)	0.00±0.000^{c}	5.63±0.850^{b}	8.08±0.904^{b}	17.93±1.533^{a}	18.61±1.656^{a}	
Propionate (mM, 0 h)	0.00±0.000	0.00±0.000	0.00±0.000	0.00±0.000	0.00±0.000	Time: $P < 0.01$; Treatment: $P < 0.01$; Time * Treatment: $P < 0.01$
Propionate (mM, 6 h)	0.00±0.000	0.00±0.000	0.00±0.000	0.00±0.000	0.00±0.000	
Propionate (mM, 12	0.00±0.000	0.00±0.000	0.00±0.000	0.00±0.000	0.00±0.000	
Propionate (mM, 24	0.00±0.000	0.04±0.036	0.03±0.033	0.07±0.038	0.00±0.000	
Propionate (mM, 48	0.00±0.000^{b}	0.00±0.000^{b}	6.31±0.769^{a}	1.06±0.112^{b}	0.35±0.247^{b}	
Butyrate (mM, 0 h)	0.00±0.000	0.00±0.000	0.00±0.000	0.00±0.000	0.00±0.000	Time: $P < 0.01$; Treatment: $P < 0.01$; Time * Treatment: $P < 0.01$
Butyrate (mM, 6 h)	0.00±0.000	0.00±0.000	0.00±0.000	0.00±0.000	0.00±0.000	
Butyrate (mM, 12 h)	0.00±0.000	0.00±0.000	0.19±0.108	0.00±0.000	0.00±0.000	
Butyrate (mM, 24 h)	0.03±0.016^{c}	0.19±0.032^{c}	0.04±0.009^{c}	0.84±0.114^{b}	1.28±0.097^{a}	
Butyrate (mM, 48 h)	0.00±0.000^{c}	0.27±0.062^{c}	0.14±0.008^{c}	3.00±0.240^{a}	1.98±0.252^{b}	
**Colon (%)**						
Acetate (mM, 0 h)	1.03±0.042	0.92±0.035	0.87±0.023	1.06±0.115	0.88±0.059	Time: $P < 0.01$; Treatment: $P < 0.01$; Time * Treatment: $P < 0.01$
Acetate (mM, 6 h)	13.62±0.930^{c}	11.65±0.504^{cd}	5.92±0.161^{d}	41.50±2.292^{a}	25.25±2.493^{b}	
Acetate (mM, 12 h)	25.15±0.708^{b}	29.18±0.298^{b}	13.53±0.189^{c}	39.38±2.062^{a}	45.18±2.509^{a}	
Acetate (mM, 24 h)	26.92±0.572^{d}	35.81±2.266^{c}	27.55±0.682^{d}	46.62±1.500^{b}	55.04±1.155^{a}	
Acetate(mM, 48 h)	25.13±0.471^{c}	35.89±0.466^{b}	33.33±1.161^{b}	49.49±1.163^{a}	56.38±3.440^{a}	
Propionate (mM, 0 h)	0.47±0.010	0.445±0.002	0.55±0.012	0.53±0.063	0.47±0.049	Time: $P < 0.01$; Treatment: $P < 0.01$; Time * Treatment: $P < 0.01$
Propionate (mM, 6 h)	11.81±0.147^{a}	9.04±0.460^{b}	4.62±0.334^{c}	1.26±0.272^{d}	3.36±0.203^{c}	
Propionate (mM, 12	22.23±0.778^{a}	21.73±0.567^{a}	14.32±0.317^{b}	2.014±0.088^{d}	5.56±0.522^{c}	
Propionate (mM, 24	29.19±1.763^{b}	31.03±3.018^{ab}	37.37±1.101^{a}	2.76±0.094^{c}	9.70±0.794^{c}	
Propionate (mM, 48	29.98±0.693^{c}	37.15±1.790^{b}	47.66±2.262^{a}	3.51±0.050^{e}	13.01±1.313^{d}	
Butyrate (mM, 0 h)	0.20±0.011	0.18±0.008	0.15±0.062	0.21±0.012	0.15±0.010	Time: $P < 0.01$; Treatment: $P < 0.01$; Time * Treatment: $P < 0.01$
Butyrate (mM, 6 h)	2.64±0.073^{a}	2.25±0.210^{ab}	0.80±0.025^{c}	1.51±0.267^{bc}	2.51±0.200^{a}	
Butyrate (mM, 12 h)	7.57±0.794^{a}	5.63±0.735^{a}	1.21±0.025^{b}	3.35±0.089^{b}	6.36±0.401^{a}	
Butyrate (mM, 24 h)	10.19±1.174^{b}	6.83±0.916^{bc}	2.90±0.604^{c}	5.45±0.324^{c}	16.18±1.226^{a}	
Butyrate (mM, 48 h)	10.31±0.386^{b}	8.70±0.548^{bc}	3.05±0.186^{d}	7.16±0.074^{c}	43.10±1.326^{a}	

[1]Data were expressed as mean ± standard error.

[a, b, c, d, e]Mean that values with different superscripts in the same row are significantly different ($P < 0.05$).
